# Supplementary material for: Screening for Metal-Chelating Activity in Potato Protein Hydrolysates Using Surface Plasmon Resonance and Peptidomics
Source: Antioxidants (Basel). 2024 Mar 13;13(3):346. doi: 10.3390/antiox13030346 (PMC10968033; doi:10.3390/antiox13030346)
Supplement: Supplementary file 1 [file antioxidants-13-00346-s001.zip › antioxidants-2874007-supplementary.pdf]

Supplementary materials

Table S1: Protein content of potato protein hydrolysate fractions and amount of protein in emulsions.

Sample	Protein content (%)	Protein in emulsion (mg)	Protein in emulsion (wt%)
AFPPH	71.8 ± 0.5	78.9	0.04
AF35	75.3 ± 1.2	82.9	0.04
AF13	73.4 ± 0.1	80.7	0.04
AF1	62.9 ± 0.2	69.1	0.03
TFPPH	68.2 ± 0.0	75.0	0.03
TF35	73.0 ± 0.3	80.3	0.04
TF13	66.8 ± 0.6	73.4	0.03

Table S2: Peptidomics data from PPHs and their fractions. Table includes all peptide-level information from the MaxQuant output data (peptides.txt) in addition to sample-wise relative signal intensity (I_rel) and predicted antioxidant scores from AnOxPePred (FRS and CHE). Table also includes peptide-wise calculations of molar AA distribution as well as intensity-weighted mean length and charge. Table is appended as separate file (Table S2.xlsx).

Table S3: Zeta potential (mV) and droplet size D[3,2] and D[4,3] in emulsions. Values are given as mean ± standard deviation. Letters (a-e) in the same column indicate a statistically significant difference between samples. * Show a significant difference in droplet size between day 1 and day 9

**Emulsion**	**D[3,2] (μm)**		**D[4,3] (μm)**		**Zeta potential (mV)**
	Day 1	Day 9	Day 1	Day 9	Day 1
**Control**	0.120±0.001^{bc}	0.120±0.000^{b}	0.185±0.001^{cd}	0.184±0.001^{bc}	(-)18.1±3.8^{a}
**EDTA**	0.121±0.001^{de}	0.122±0.000^{d,*}	0.190±0.001^{e}	0.192±0.000^{de}	(-)19.6±2.8^{a}
**AFPPH**	0.118±0.000^{a}	0.119±0.000^{a,*}	0.180±0.000^{a}	0.181±0.002^{a}	(-)15.4±3.2^{a}
**AF35**	0.119±0.000^{b}	0.120±0.001^{ab}	0.182±0.000^{ab}	0.182±0.001^{ab}	(-)15.1±1.8^{a}
**AF13**	0.121±0.000^{e}	0.123±0.001^{d}	0.191±0.001^{e}	0.194±0.001^{e}	(-)14.1±1.0^{a}
**AF1**	0.120±0.000^{cd}	0.120±0.000^{bc,*}	0.187±0.001^{d}	0.187±0.000^{c}	(-)14.3±1.6^{a}
**TFPPH**	0.120±0.000^{b}	0.122±0.001^{cd}	0.189±0.000^{e}	0.190±0.001^{d}	(-)14.1±0.8^{a}
**TF35**	0.120±0.001^{bc}	0.120±0.001^{ab}	0.184±0.000^{bc}	0.183±0.000^{ab}	(-)13.3±1.3^{a}
**TF13**	0.119±0.001^{d}	0.120±0.000^{b,*}	0.183±0.001^{bc}	0.184±0.001^{abc}	(-)12.4±1.0^{a}

Table S4: alpha-tocopherol consumption in % during 9 days of storage

	Day 3	Day 6	Day 9
Control	96.2	100	100
EDTA	19.5	23.4	21.7
AFPPH	22	78	77.7
AF35	26.2	50.8	71
AF13	21.9	50	57.8
Af1	14.5	40.3	54.3
TFPPH	35.5	74.2	81.5
TF35	32.3	54.1	78.9

TF13	26.9	41.2	63.7

Table S5 ***1-penten-3-ol (ng/g).*** *Letters (a-c) indicate a statistically significant difference between emulsions (P<0.05). Letters (v-z) indicate a statistically significant difference between days (P<0.05). Numbers (1-4) indicate a statistically significant difference between emulsions (without the control) on day 9*

Sample name	Day 0	Day 3	Day 6	Day 9
**Control**	34 ± 6.3^{c,v}	317.4 ± 27.3^{d,x}	688.7 ± 58.1^{c,y}	1266.4 ± 109.8^{c,z}
**EDTA**	11.2 ± 3.5^{a,v}	47.1 ± 6.9^{a,vx}	101.6 ± 23.8^{a,xy}	131.7 ± 39.3^{a,y,1}
**AFPPH**	25.8 ± 2.2^{abc,v}	136.8 ± 21.2^{b,x}	315.4 ± 36.2^{b,y}	505.2 ±32.1^{b,z,3}
**AF35**	30.6 ± 3.6^{bc,v}	156.3 ± 9^{bc,x}	273.9 ± 16.2^{b,y}	463 ± 37.4^{b,z,23}
**AF13**	27.6 ± 2.9^{bc,v}	143 ± 3.3^{b,x}	315.5 ± 37.4^{b,y}	363.5 ± 13.5^{b,y,2}
**AF1**	21.3 ± 11.9^{abc,v}	113.4 ± 19.7^{b,x}	280.7 ± 31.4^{b,y}	372.2 ± 46.7^{b,z,2}
**TFPPH**	29.6 ± 2.6^{bc,v}	206.3 ± 5.7^{c,x}	385.5 ± 70.9^{b,y}	470.2 ± 8.1^{b,y,23}
**TF35**	18.6 ± 5.7^{ab,v}	143 ± 6^{b,x}	324.5 ± 41.3^{b,y}	426.6 ± 78.4^{b,y,23}
**TF13**	18.8 ± 1.1^{abc,v}	134.9 ± 32.6^{b,x}	269.5 ± 43.3^{b,y}	390.3 ± 18.7^{b,z,2}

Table S6 ***Hexanal(ng/g).*** *Letters (a-d) indicate statistically significant difference between emulsions (P<0.05). Letters (v-z) indicate statistically significant difference between days (P<0.05). Numbers (1-4) indicate a statistically significant difference between emulsions (without the control) on day 9*

Sample name	Day 0	Day 3	Day 6	Day 9
**Control**	8.8 ± 1.7^{ab,v}	30 ± 1.8^{c,vx}	64.4 ± 7.1^{c,x}	161.4 ± 36.9^{b,y}
**EDTA**	5.4 ± 1.7^{a,v}	7.6 ± 0.8^{a,v}	10.9 ± 2.9^{a,v}	12.8 ± 5.9^{a,v,1}
**AFPPH**	10.8 ± 0.7^{b,v}	16.7 ± 1.7^{b,x}	24.5 ± 2.1^{b,y}	36.5 ± 3.1^{a,z,34}
**AF35**	10.2 ± 0.7^{b,v}	15.4 ± 0.3^{b,vx}	20.2 ± 1.1^{ab,x}	27.1 ± 4.7^{a,y,234}
**AF13**	10.1 ± 0.5^{b,v}	15.7 ± 0.8^{b,vx}	21 ± 3.3^{ab,xy}	24.3 ± 5^{a,y,12}
**AF1**	7.8 ± 3.6^{ab,v}	14.6 ± 1.2^{b,vx}	19.6 ± 4.2^{ab,xy}	26.9 ± 1.5^{a,y,23}
**TFPPH**	11.6 ± 0.4^{b,v}	16.6 ± 1.4^{b,vx}	22.4 ± 5.8^{ab,x}	38.7 ± 1.7^{a,y,4}
**TF35**	8.9 ± 1.3^{ab,v}	15.6 ± 0.4^{b,vx}	21.2 ± 3.7^{ab,xy}	29.7 ± 6.1^{a,y,234}
**TF13**	8.4 ± 0.9^{ab,v}	13.5 ± 2.2^{b,vx}	17 ± 4.3^{ab,x}	30 ± 2.1^{a,y,234}

Table S7 *3-methyl-butanal (ng/g). Letters (a-e) indicate statistically significant difference between emulsions (P<0.05). Letters (v-y) indicate statistically significant difference between days (P<0.05). Numbers (1-4) indicate a statistically significant difference between emulsions (without the control) on day 9*

Sample name	Day 0	Day 3	Day 6	Day 9
**Control**	$0^{a,v}$	$0^{a,v}$	$0^{a,v}$	$0^{a,v}$
**EDTA**	$0^{a,v}$	$0^{a,v}$	$0^{a,v}$	$0^{a,v}$
**AFPPH**	$2.9 \pm 0.3^{b,v}$	$4.2 \pm 0.8^{b,v}$	$9.4 \pm 1.8^{b,x}$	$15.9 \pm 1.6^{bcd,y,12}$
**AF35**	$3 \pm 0.5^{b,v}$	$4.6 \pm 0.4^{b,v}$	$6.4 \pm 0.6^{ab,vx}$	$9.3 \pm 2.5^{b,x,1}$
**AF13**	$2.2 \pm 0.2^{b,v}$	$4.7 \pm 0.1^{b,vx}$	$7.7 \pm 1.9^{b,xy}$	$10 \pm 1.6^{b,y,1}$
**AF1**	$2.5 \pm 1.5^{b,v}$	$6.7 \pm 1.4^{b,vx}$	$13.5 \pm 3.6^{b,xy}$	$18.4 \pm 3.5^{cd,y,23}$
**TFPPH**	$3.8 \pm 0.4^{b,v}$	$6.6 \pm 0.6^{b,vx}$	$11 \pm 4.4^{b,x}$	$18 \pm 2^{cde,y,23}$
**TF35**	$2.4 \pm 0.7^{b,v}$	$4.7 \pm 0.4^{b,vx}$	$9 \pm 2.5^{b,xy}$	$11.4 \pm 3.9^{bc,y,12}$
**TF13**	$2.5 \pm 0.5^{b,v}$	$6.4 \pm 2.4^{b,vx}$	$11.2 \pm 4.1^{b,x}$	$24.6 \pm 2.5^{e,y,3}$

Table S8 *2-ethylfuran (ng/g). Letters (a-b) indicate statistically significant difference between emulsions (P<0.05). Letters (v-y) indicate statistically significant difference between days (P<0.05).*

Sample name	Day 0	Day 3	Day 6	Day 9
**Control**	$1.6 \pm 0.4^{b,v}$	$20.1 \pm 3.9^{b,vx}$	$63.5 \pm 11.1^{b,x}$	$187.4 \pm 39.1^{b,y}$
**EDTA**	$1 \pm 0.2^{a,v}$	$7.3 \pm 1.5^{a,vx}$	$14.9 \pm 5.3^{a,vx}$	$21 \pm 12.9^{a,x}$
**AFPPH**	$0.7 \pm 0.1^{ab,v}$	$10 \pm 2.4^{a,v}$	$29.1 \pm 5.1^{a,x}$	$44.1 \pm 7.9^{a,y}$
**AF35**	$0.8 \pm 0.1^{a,v}$	$11.7 \pm 1.6^{a,vx}$	$22.9 \pm 2.7^{a,xy}$	$30.6 \pm 10.9^{a,y}$
**AF13**	$0.8 \pm 0.2^{a,v}$	$12.6 \pm 0.5^{a,vx}$	$21.9 \pm 5.8^{a,xy}$	$29.5 \pm 8.9^{a,y}$
**AF1**	$0.8 \pm 0.3^{a,v}$	$11.8 \pm 2.9^{a,vx}$	$23.7 \pm 8.8^{a,xy}$	$30.3 \pm 6.1^{a,y}$
**TFPPH**	$1.1 \pm 0.1^{a,v}$	$13.7 \pm 1^{ab,vx}$	$26.1 \pm 13.8^{a,xy}$	$45.2 \pm 5.2^{a,y}$
**TF35**	$0.6 \pm 0.1^{a,v}$	$11.5 \pm 0.6^{a,vx}$	$24 \pm 7.6^{a,xy}$	$31 \pm 11.6^{a,y}$
**TF13**	$0.7 \pm 0.1^{a,v}$	$12.2 \pm 4.2^{a,v}$	$18.6 \pm 11.8^{a,v}$	$44.9 \pm 6.4^{a,x}$

Table S9 ***Pentanal (ng/g).*** *Letters (a-c) indicate statistically significant difference between emulsions (P<0.05). Letters (v-y) indicate statistically significant difference between days (P<0.05).*

Sample name	Day 0	Day 3	Day 6	Day 9
**Control**	$9.4 \pm 1.7^{ab,v}$	$21.8 \pm 2.1^{c,vx}$	$50.6 \pm 3.9^{c,x}$	$136.4 \pm 24.1^{c,y}$
**EDTA**	$5.5 \pm 1.8^{a,v}$	$6.6 \pm 0.9^{a,v}$	$8.9 \pm 2.6^{a,v}$	$10 \pm 4.6^{a,v}$
**AFPPH**	$12.6 \pm 1.3^{b,v}$	$15.4 \pm 2.1^{b,v}$	$22.9 \pm 2.5^{b,x}$	$35.5 \pm 3.6^{b,y}$
**AF35**	$11.9 \pm 1.5^{ab,v}$	$16.2 \pm 0.6^{b,vx}$	$18.8 \pm 0.8^{ab,x}$	$27.3 \pm 4.7^{ab,y}$
**AF13**	$10.7 \pm 0.8^{ab,v}$	$16.5 \pm 0.6^{b,vx}$	$21.8 \pm 3.6^{b,xy}$	$24.6 \pm 3.8^{ab,y}$
**AF1**	$10.1 \pm 5.4^{ab,v}$	$15.4 \pm 2.1^{b,vx}$	$21.4 \pm 4.3^{b,xy}$	$27.9 \pm 3.3^{ab,y}$
**TFPPH**	$15.8 \pm 1.2^{b,v}$	$19.1 \pm 0.9^{bc,v}$	$23.9 \pm 6.6^{b,v}$	$37.6 \pm 1.8^{b,x}$
**TF35**	$10.8 \pm 2.1^{ab,v}$	$15.5 \pm 0.4^{b,vx}$	$23.8 \pm 5.6^{b,xy}$	$28.8 \pm 6.5^{ab,y}$
**TF13**	$10.8 \pm 1.7^{ab,v}$	$15 \pm 3.8^{b,v}$	$19 \pm 4.9^{ab,v}$	$31.4 \pm 2.1^{ab,x}$

Table S10 ***Heptanal (ng/g).*** *Letters (a-d) indicate statistically significant difference between emulsions (P<0.05). Letters (v-x) indicate statistically significant difference between days (P<0.05).*

Sample name	Day 0	Day 3	Day 6	Day 9
**Control**	$13 \pm 2.6^{ab,v}$	$27.1 \pm 1.3^{c,v}$	$54.3 \pm 5.9^{d,v}$	$147.7 \pm 37.7^{b,x}$
**EDTA**	$9.2 \pm 1.9^{a,v}$	$8.7 \pm 1.1^{a,v}$	$12.1 \pm 3^{a,v}$	$11.2 \pm 5^{a,v}$
**AFPPH**	$16.1 \pm 2.4^{b,v}$	$18.3 \pm 1.7^{b,vx}$	$23.5 \pm 1.4^{b,x}$	$34.1 \pm 2.9^{a,y}$
**AF35**	$14.5 \pm 1.1^{ab,v}$	$19.2 \pm 4.5^{b,v}$	$21.1 \pm 1^{ab,vx}$	$27.3 \pm 3.7^{a,x}$
**AF13**	$14.9 \pm 0.3^{ab,v}$	$16.3 \pm 0.8^{b,vx}$	$20.3 \pm 3.1^{ab,vx}$	$23.2 \pm 5.1^{a,x}$
**AF1**	$12.7 \pm 4.8^{ab,v}$	$16.6 \pm 1.9^{b,v}$	$20.2 \pm 4.1^{ab,vx}$	$25.8 \pm 1.2^{a,x}$
**TFPPH**	$16.6 \pm 0.3^{b,v}$	$18 \pm 1.1^{b,v}$	$23 \pm 5.2^{ab,v}$	$39 \pm 2^{a,x}$
**TF35**	$13.4 \pm 1.6^{ab,v}$	$17.2 \pm 1.2^{b,v}$	$36.4 \pm 5^{c,x}$	$30.3 \pm 5.2^{a,x}$
**TF13**	$13.6 \pm 1.4^{ab,v}$	$15.8 \pm 1.8^{b,v}$	$19.6 \pm 4.2^{ab,v}$	$21 \pm 18.2^{a,v}$

Table S11 ***Trans-2-heptenal (ng/g)***. *Letters (a-c) indicate statistically significant difference between emulsions (P<0.05). Letters (v-z) indicate statistically significant difference between days (P<0.05).*

Sample name	Day 0	Day 3	Day 6	Day 9
**Control**	$0.3 \pm 0.2^{b,v}$	$1.2 \pm 0.1^{d,v}$	$6.7 \pm 1.3^{b,x}$	$8.2 \pm 0.3^{c,x}$
**EDTA**	$0.1 \pm 0.03^{a,v}$	$0.1 \pm 0.1^{a,v}$	$0.4 \pm 0.3^{a,v}$	$0.3 \pm 0.2^{a,v}$
**AFPPH**	$0.1 \pm 0.01^{a,v}$	$0.2 \pm 0.1^{ab,v}$	$0.5 \pm 0.1^{a,x}$	$0.8 \pm 0.2^{ab,y}$
**AF35**	$0.1 \pm 0.02^{a,v}$	$0.3 \pm 0.1^{bc,x}$	$0.7 \pm 0.1^{a,y}$	$0.8 \pm 0.1^{ab,y}$
**AF13**	$0.1 \pm 0.04^{a,v}$	$0.2 \pm 0.01^{abc,v}$	$0.4 \pm 0.1^{a,x}$	$0.6 \pm 0.05^{a,y}$
**AF1**	$0.1 \pm 0.03^{a,v}$	$0.2 \pm 0.02^{ab,v}$	$0.3 \pm 0.05^{a,x}$	$0.6 \pm 0.05^{a,y}$
**TFPPH**	$0.1 \pm 0.04^{a,v}$	$0.4 \pm 0.02^{c,vx}$	$0.6 \pm 0.1^{a,x}$	$1.4 \pm 0.2^{b,y}$
**TF35**	$0.1 \pm 0.01^{a,v}$	$0.3 \pm 0.01^{bc,v}$	$0.4 \pm 0.2^{a,v}$	$1.3 \pm 0.3^{b,x}$
**TF13**	$0.1 \pm 0.02^{a,v}$	$0.2 \pm 0.03^{ab,x}$	$0.3 \pm 0.06^{a,y}$	$0.6 \pm 0.01^{a,z}$

Table S12 ***1-octen-3-ol (ng/g)***. *Letters (a-e) indicate statistically significant difference between emulsions (P<0.05). Letters (v-z) indicate statistically significant difference between days (P<0.05).*

Sample name	Day 0	Day 3	Day 6	Day 9
**Control**	$1.6 \pm 0.3^{c,v}$	$10.3 \pm 0.1^{e,x}$	$23.3 \pm 0.4^{e,y}$	$45.7 \pm 4.3^{d,z}$
**EDTA**	$0.5 \pm 0.2^{a,v}$	$1 \pm 0.5^{a,v}$	$2 \pm 0.4^{a,v}$	$1.6 \pm 1.6^{a,v}$
**AFPPH**	$1 \pm 1.2^{ab,v}$	$4.2 \pm 0.4^{c,x}$	$9.2 \pm 0.1^{c,y}$	$15.2 \pm 0.3^{c,z}$
**AF35**	$1.1 \pm 0.03^{b,v}$	$3.7 \pm 0.3^{c,x}$	$8 \pm 0.5^{bc,y}$	$14.9 \pm 0.6^{c,z}$
**AF13**	$1.2 \pm 0.1^{bc,v}$	$3.4 \pm 0.3^{bc,x}$	$8.5 \pm 0.6^{bc,y}$	$9.7 \pm 0.4^{b,z}$
**AF1**	$1 \pm 0.2^{b,v}$	$2.8 \pm 0.2^{b,x}$	$7.4 \pm 1.1^{bc,y}$	$10.7 \pm 0.6^{bc,z}$
**TFPPH**	$1.1 \pm 0.1^{b,v}$	$5.6 \pm 0.3^{d,x}$	$11.8 \pm 1.3^{d,y}$	$14.9 \pm 0.8^{c,z}$
**TF35**	$0.8 \pm 0.1^{ab,v}$	$3.9 \pm 0.3^{c,x}$	$7.9 \pm 0.6^{bc,y}$	$14.3 \pm 1.1^{bc,z}$
**TF13**	$0.9 \pm 0.1^{ab,v}$	$3.6 \pm 0.03^{bc,x}$	$6.7 \pm 1.2^{b,y}$	$9.8 \pm 0.6^{b,z}$

Table S13 ***Benzaldehyd (ng/g).*** *Letters (a-e) indicate statistically significant difference between emulsions (P<0.05). Letters (v-z) indicate statistically significant difference between days (P<0.05).*

Sample name	Day 0	Day 3	Day 6	Day 9
**Control**	$1.7 \pm 0.3^{ab,v}$	$2.2 \pm 0.2^{a,v}$	$3.1 \pm 0.3^{a,v}$	$6.7 \pm 3.8^{a,v}$
**EDTA**	$1.1 \pm 0.5^{a,v}$	$2.1 \pm 0.6^{a,vx}$	$3 \pm 0.7^{a,x}$	$2.2 \pm 0.7^{a,vx}$
**AFPPH**	$3.3 \pm 0.1^{bc,v}$	$7.1 \pm 0.4^{bc,x}$	$11.3 \pm 0.3^{ab,y}$	$17 \pm 0.9^{bc,z}$
**AF35**	$2.7 \pm 0.1^{abc,v}$	$5.9 \pm 0.4^{b,x}$	$9.4 \pm 0.3^{ab,y}$	$15 \pm 0.6^{bc,z}$
**AF13**	$2.3 \pm 2^{abc,v}$	$6.7 \pm 0.7^{bc,v}$	$11.9 \pm 1.7^{ab,x}$	$13 \pm 2.4^{b,x}$
**AF1**	$2.5 \pm 0.4^{abc,v}$	$5.9 \pm 0.6^{b,x}$	$12.2 \pm 1.9^{ab,y}$	$16.9 \pm 0.9^{bc,z}$
**TFPPH**	$4 \pm 0.06^{c,v}$	$9.4 \pm 0.3^{d,x}$	$15.8 \pm 1.5^{b,y}$	$19.6 \pm 0.8^{c,z}$
**TF35**	$3.2 \pm 0.3^{bc,v}$	$7.4 \pm 0.3^{c,vx}$	$17.1 \pm 10.2^{b,x}$	$18.8 \pm 0.8^{c,x}$
**TF13**	$2.9 \pm 0.2^{abc,v}$	$7.9 \pm 0.2^{c,x}$	$11 \pm 1.6^{ab,y}$	$19.1 \pm 0.2^{c,z}$

Table S14 ***Octanal (ng/g).*** *Letters (a-d) indicate statistically significant difference between emulsions (P<0.05). Letters (v-y) indicate statistically significant difference between days (P<0.05).*

Sample name	Day 0	Day 3	Day 6	Day 9
**Control**	$7.4 \pm 1.6^{ab,v}$	$17.9 \pm 0.5^{c,vx}$	$34.2 \pm 2^{d,x}$	$84.8 \pm 18.4^{b,y}$
**EDTA**	$4.8 \pm 1.6^{a,v}$	$4.8 \pm 0.9^{a,v}$	$7.2 \pm 1.4^{a,v}$	$6.6 \pm 2.5^{a,v}$
**AFPPH**	$7.9 \pm 1.1^{b,v}$	$9.9 \pm 0.7^{b,v}$	$13 \pm 0.8^{b,x}$	$20.2 \pm 1.4^{a,y}$
**AF35**	$6.8 \pm 0.6^{ab,v}$	$9.6 \pm 1.9^{b,x}$	$11.6 \pm 0.4^{ab,x}$	$16.6 \pm 0.9^{a,y}$
**AF13**	$8 \pm 0^{b,v}$	$8.3 \pm 0.5^{b,v}$	$11.7 \pm 1.7^{ab,x}$	$13.5 \pm 1.6^{a,x}$
**AF1**	$6.9 \pm 1.6^{ab,v}$	$8.9 \pm 0.8^{b,vx}$	$11.8 \pm 2.1^{ab,x}$	$16.3 \pm 0.8^{a,y}$
**TFPPH**	$7.5 \pm 0.5^{ab,v}$	$9.9 \pm 0.4^{b,v}$	$13.5 \pm 2.2^{bc,x}$	$22.9 \pm 0.8^{a,y}$
**TF35**	$6.5 \pm 0.8^{ab,v}$	$9.3 \pm 0.5^{b,v}$	$18.1 \pm 1.6^{c,x}$	$19.7 \pm 2.3^{a,x}$
**TF13**	$6.6 \pm 0.1^{ab,v}$	$8.2 \pm 0.6^{b,v}$	$10.7 \pm 1.6^{ab,x}$	$17.3 \pm 0.7^{a,y}$

Table S15 ***2,4-heptadienal (ng/g)***. *Letters (a-d) indicate statistically significant difference between emulsions (P<0.05). Letters (v-y) indicate statistically significant difference between days (P<0.05).*

Sample name	Day 0	Day 3	Day 6	Day 9
**Control**	32 ± 6.9^{b,v}	262.6 ± 15.3^{c,x}	486.4 ± 40.6^{c,y}	653.2 ± 7^{d,z}
**EDTA**	7 ± 4.1^{a,v}	24.1 ± 12.9^{a,vx}	47.6 ± 9^{a,vx}	68.6 ± 36.6^{a,x}
**AFPPH**	12.1 ± 0.1^{a,v}	31.5 ± 2.8^{ab,v}	74.2 ± 15.3^{ab,x}	171.6 ± 3.6^{b,y}
**AF35**	10 ± 1.7^{a,v}	29.6 ± 6.3^{ab,v}	86.8 ± 4.4^{ab,x}	174 ± 19.6^{b,y}
**AF13**	8.3 ± 3.8^{a,v}	20.9 ± 2.7^{a,v}	53.4 ± 1.3^{a,x}	102.8 ± 9.8^{a,y}
**AF1**	6.2 ± 1^{a,v}	17.3 ± 4^{a,v}	40.3 ± 6.5^{a,x}	100.6 ± 13.7^{a,y}
**TFPPH**	14.8 ± 4^{a,v}	48.1 ± 11.1^{b,v}	112.8 ± 21.4^{b,x}	238.3 ± 11.3^{c,y}
**TF35**	9.4 ± 2.8^{a,v}	32.8 ± 2.8^{ab,vx}	62.8 ± 16.8^{ab,x}	208.6 ± 26.6^{bc,y}
**TF13**	7.5 ± 0.9^{a,v}	22.1 ± 4.8^{a,vx}	47.1 ± 10.8^{a,x}	111 ± 9.7^{a,y}

Table S16 ***2,4-decadienal (ng/g)***. *Letters (a-d) indicate statistically significant difference between emulsions (P<0.05). Letters (v-y) indicate statistically significant difference between days (P<0.05).*

Sample name	Day 0	Day 3	Day 6	Day 9
**Control**	0.8 ± 0.2^{a,v}	14.8 ± 3.4^{b,v}	38.1 ± 7.3^{b,x}	69.2 ± 7.2^{d,y}
**EDTA**	0.3 ± 0.3^{a,v}	0.7 ± 0.7^{a,v}	1.7 ± 0,1^{a,v}	2.4 ± 1.5^{a,v}
**AFPPH**	0.6 ± 0.1^{a,v}	2.1 ± 0.4^{a,vx}	4.3 ± 1.7^{a,x}	12.5 ± 2.1^{abc,y}
**AF35**	0.4 ± 0.2^{a,v}	2 ± 0.9^{a,v}	6.2 ± 1.1^{a,v}	15.6 ± 5.5^{bc,x}
**AF13**	0.5 ± 0.2^{a,v}	1.4 ± 0.6^{a,vx}	4.2 ± 0.6^{a,x}	8.3 ± 2.3^{ab,y}
**AF1**	0.6 ± 0.3^{a,v}	1.2 ± 0.5^{a,v}	2.8 ± 1^{a,v}	8.3 ± 2.6^{abc,x}
**TFPPH**	0.5 ± 0.2^{a,v}	3.2 ± 1.4^{a,v}	8.3 ± 2.6^{a,x}	18.4 ± 0.5^{c,y}
**TF35**	0.4 ± 0.2^{a,v}	1.9 ± 0.3^{a,v}	3.6 ± 1.6^{a,v}	17.2 ± 3.2^{bc,x}
**TF13**	0.3 ± 0.1^{a,v}	1.3 ± 0.5^{a,vx}	3.9 ± 0.6^{a,x}	7.4 ± 1.3^{ab,y}

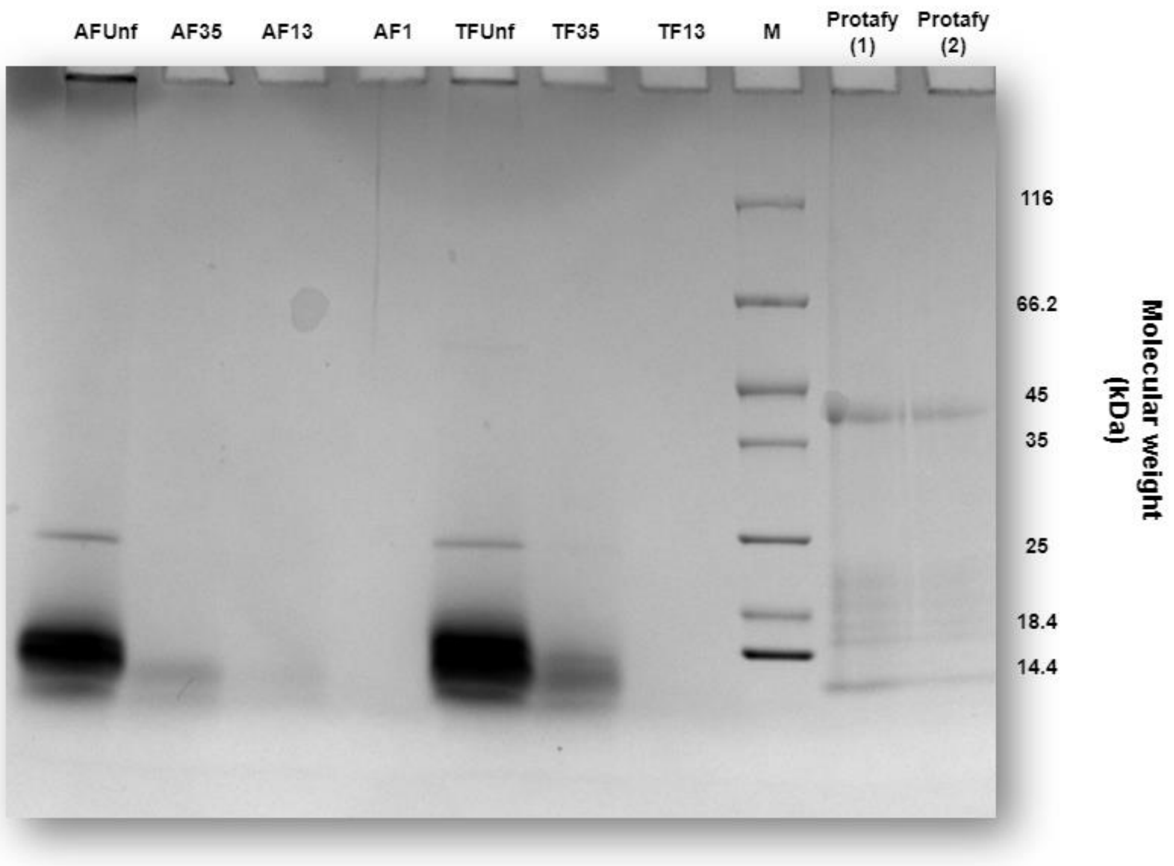

Figure S1: SDS-PAGE analysis of crude supernatant and fractionated PPHs following sequential hydrolysis with Alcalase and Flavourzyme or Trypsin and Flavourzyme. The substrate (Protafy 130) is included as reference.

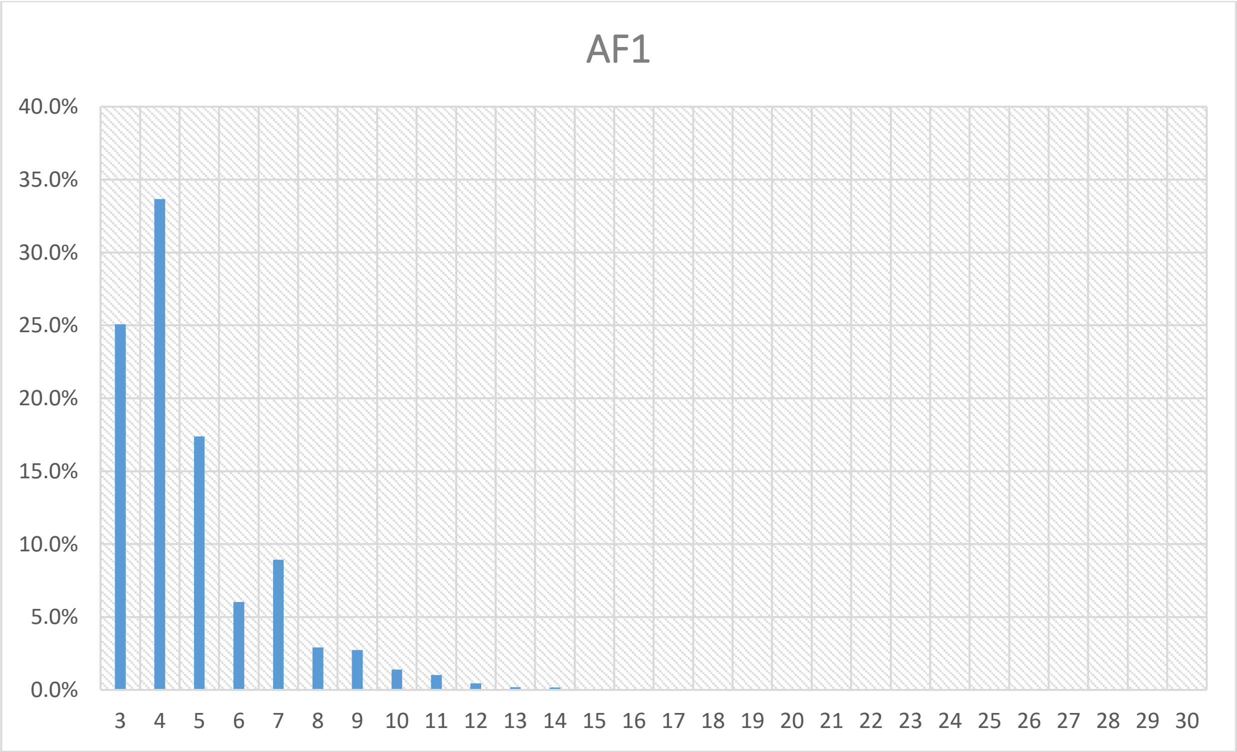

Figure S2: Relative, intensity-weighted length distribution (n = 3-30) of peptides identified in fraction AF1 from peptidomics analysis by unspecific analysis of LC-MS/MS data in MaxQuant.

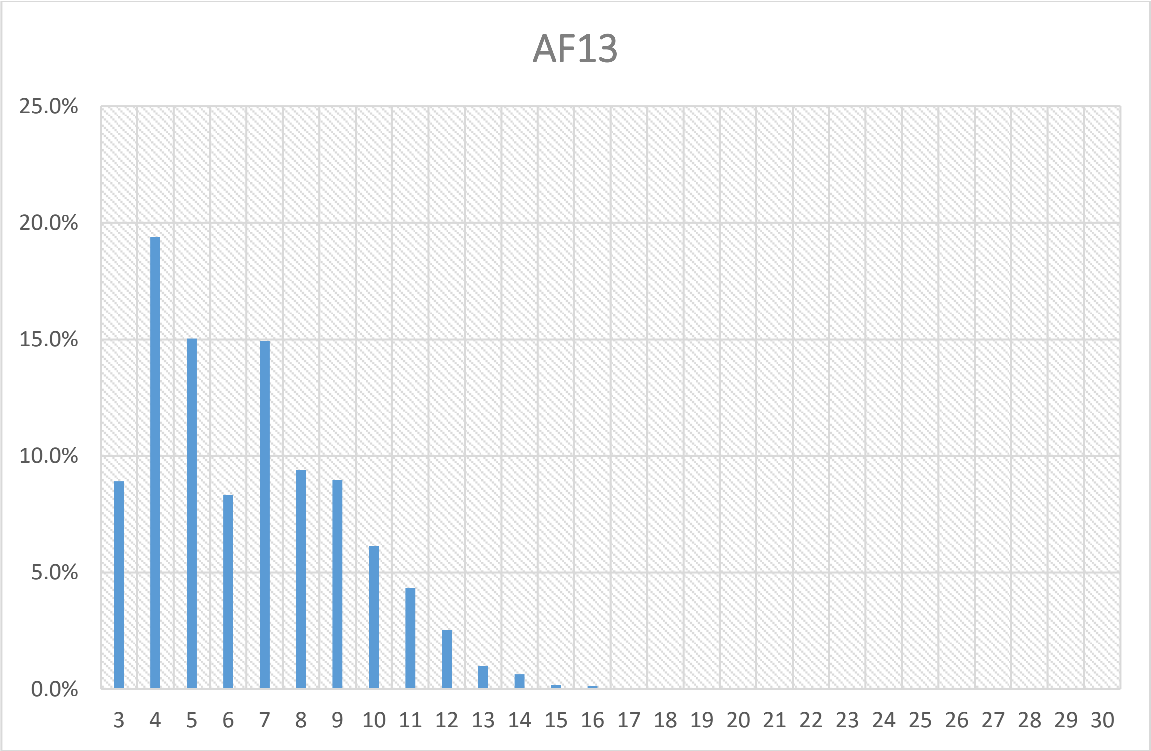

Figure S3: Relative, intensity-weighted length distribution (n = 3-30) of peptides identified in fraction AF35 from peptidomics analysis by unspecific analysis of LC-MS/MS data in MaxQuant.

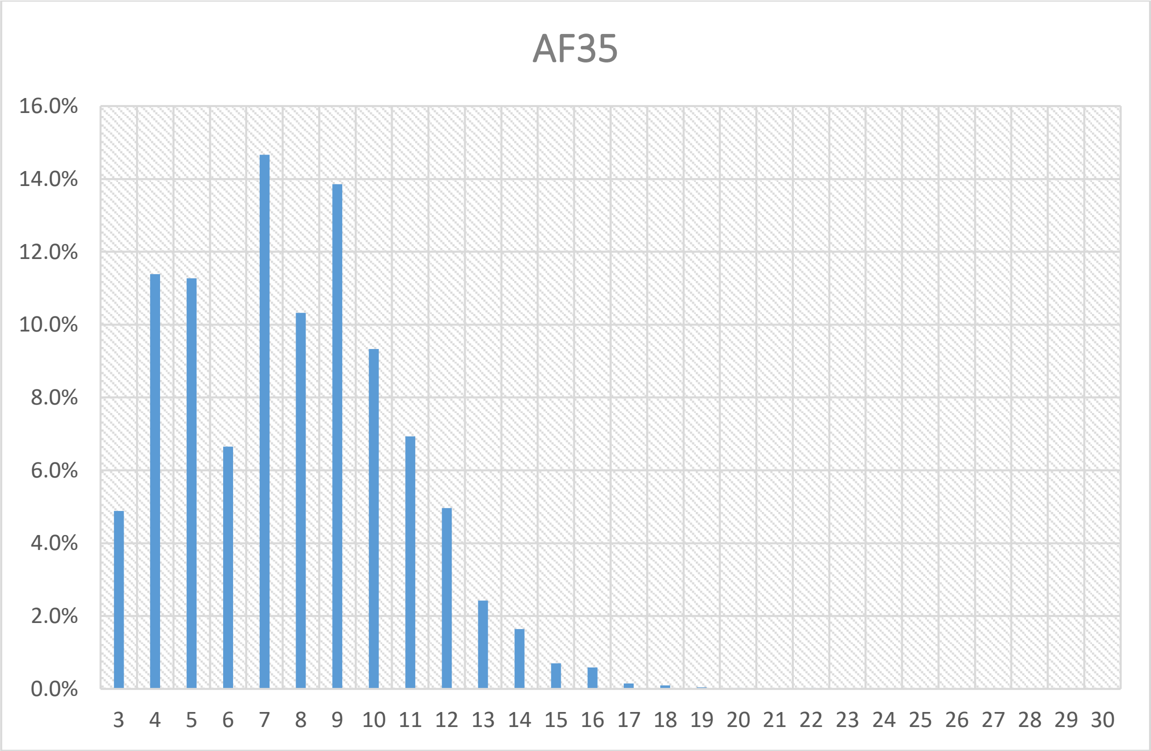

Figure S4: Relative, intensity-weighted length distribution (n = 3-30) of peptides identified in fraction AF35 from peptidomics analysis by unspecific analysis of LC-MS/MS data in MaxQuant.

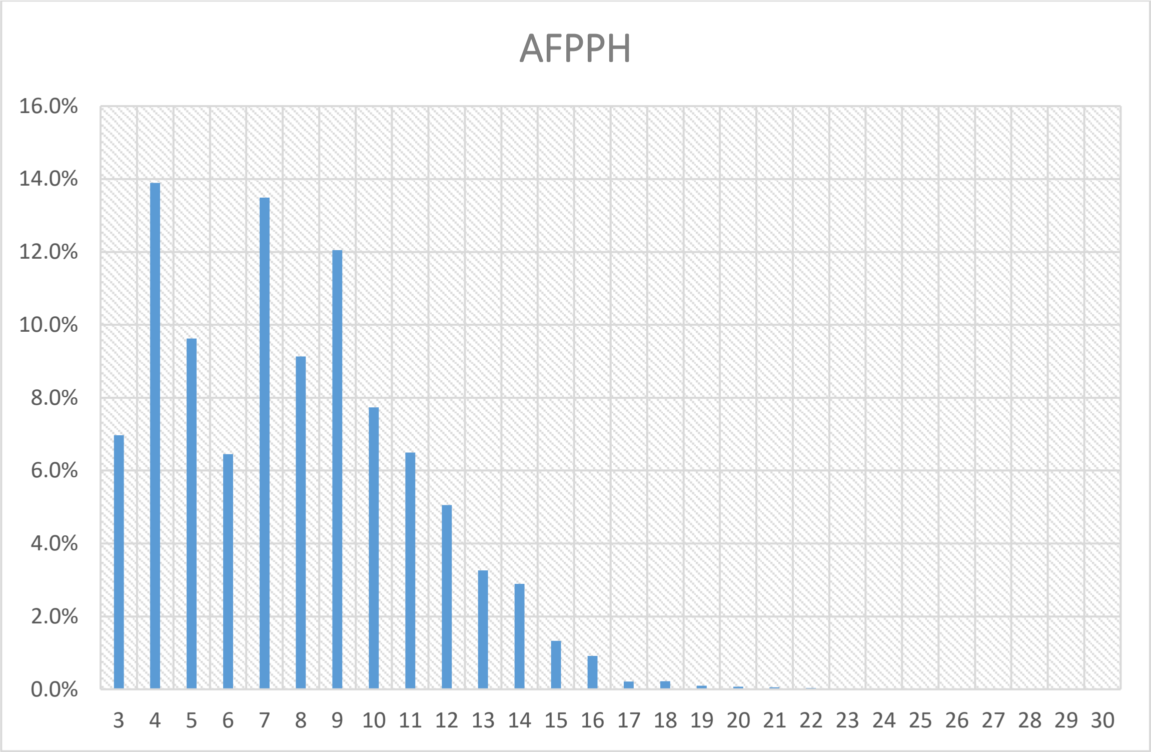

Figure S5: Relative, intensity-weighted length distribution (n = 3-30) of peptides identified in unfractionated AFPPH from peptidomics analysis by unspecific analysis of LC-MS/MS data in MaxQuant.

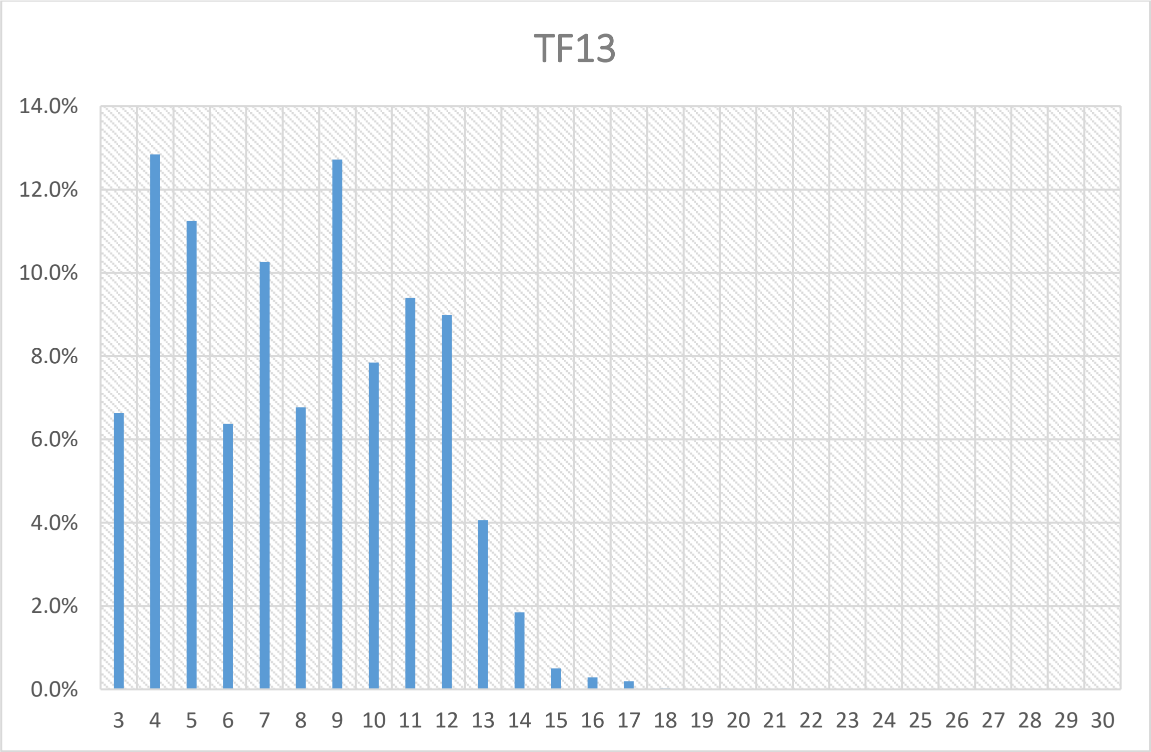

Figure S6: Relative, intensity-weighted length distribution (n = 3-30) of peptides identified in fraction TF13 from peptidomics analysis by unspecific analysis of LC-MS/MS data in MaxQuant.

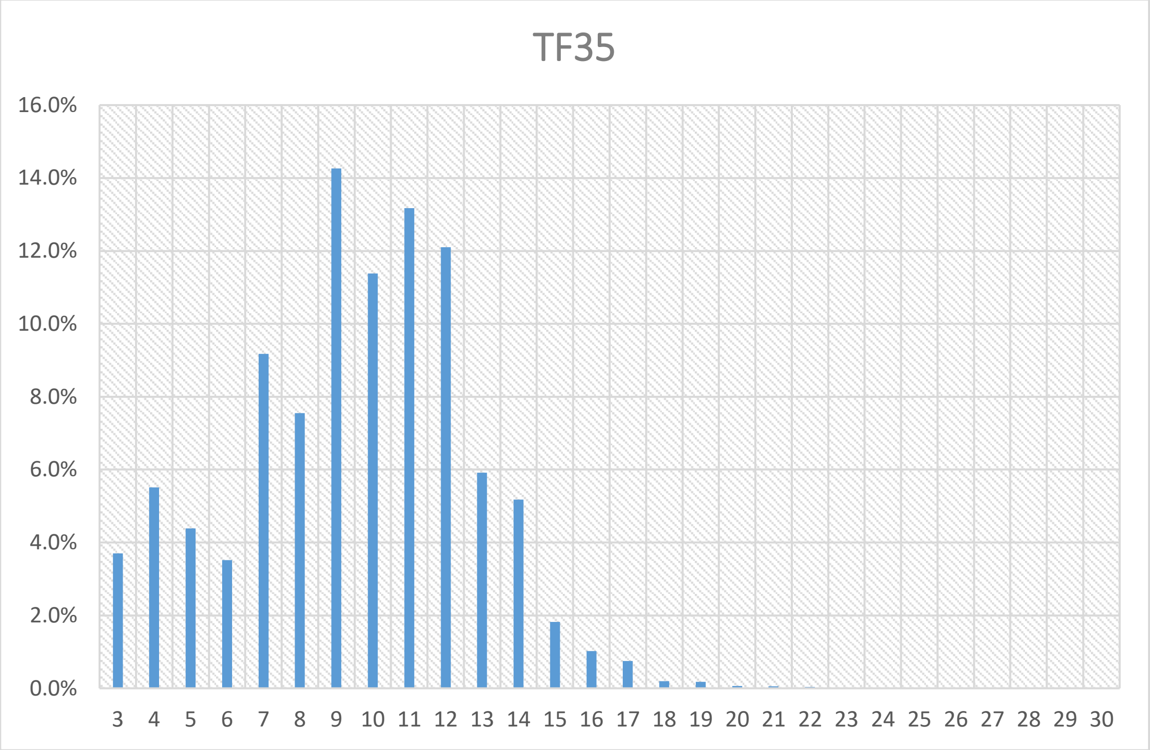

Figure S7: Relative, intensity-weighted length distribution (n = 3-30) of peptides identified in fraction TF35 from peptidomics analysis by unspecific analysis of LC-MS/MS data in MaxQuant.

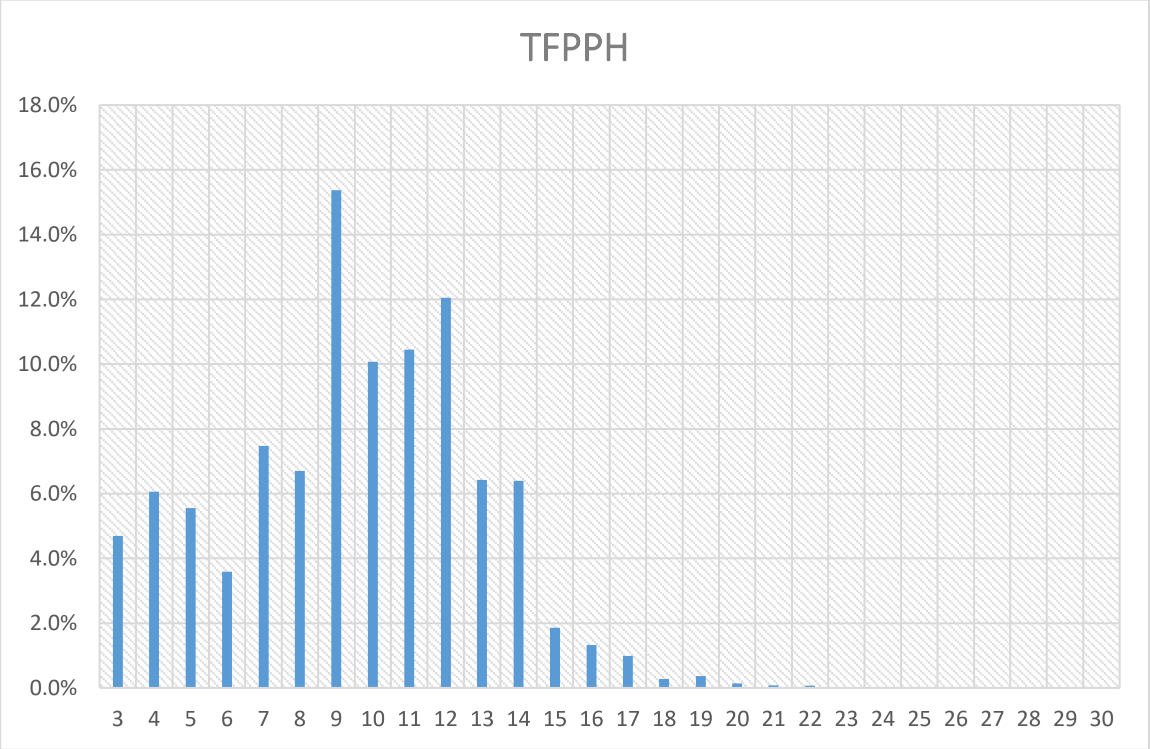

Figure S8: Relative, intensity-weighted length distribution (n = 3-30) of peptides identified in unfractionated TFPPH from peptidomics analysis by unspecific analysis of LC-MS/MS data in MaxQuant.

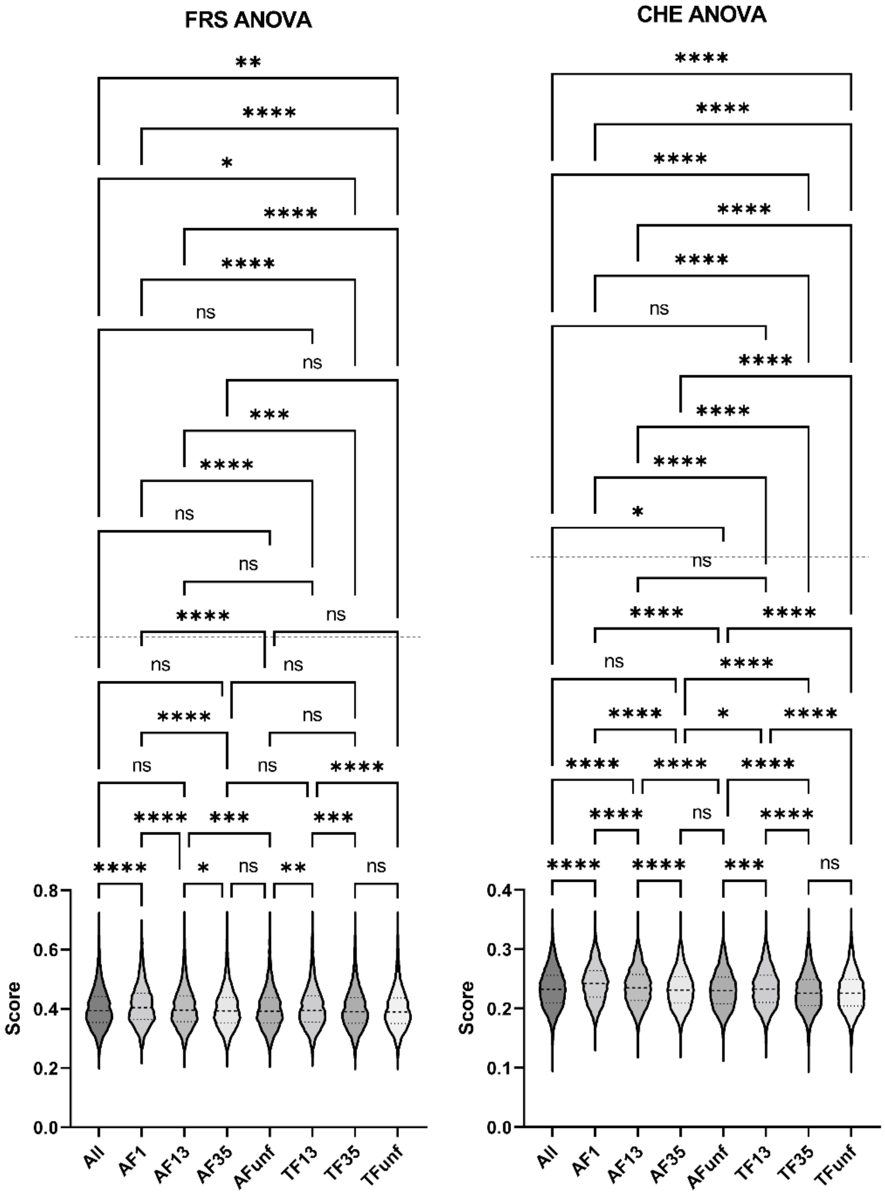

Figure S9: Depiction of one-way ANOVA and Tukey test for FRS (A) and CHE (B) score distribution by pairwise comparison. Significance level is indicated by adjusted p value as: not significant (ns), < 0.0332 (*), < 0.0021 (**), < 0.0002 (***), and < 0.0001 (****).

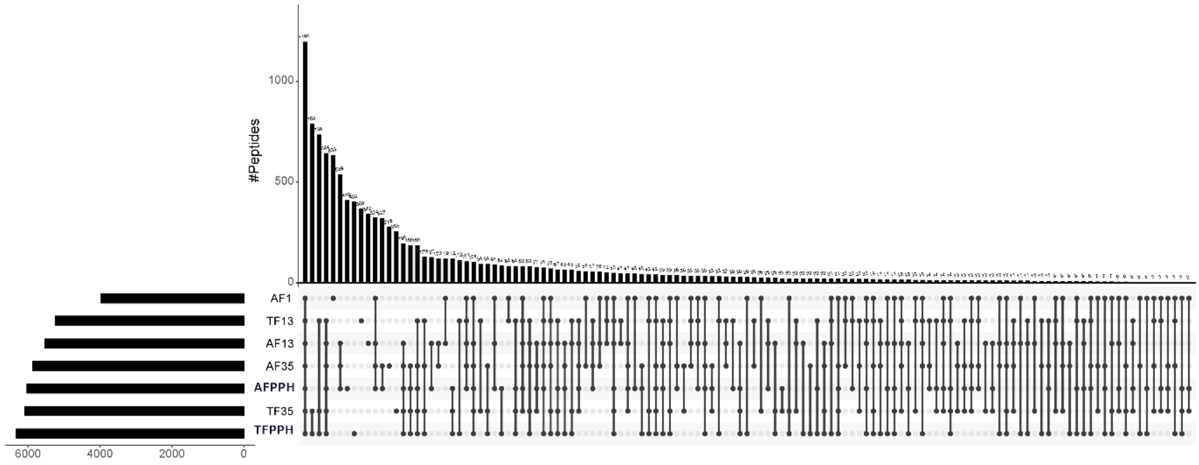

Figure S10: Full UpSet plot showing overlap of identified peptides between PPH fractions and indicating the arithmetic magnitude of the intersects (i.e., number of shared peptides) as well as the total number of peptide IDs within each fraction.

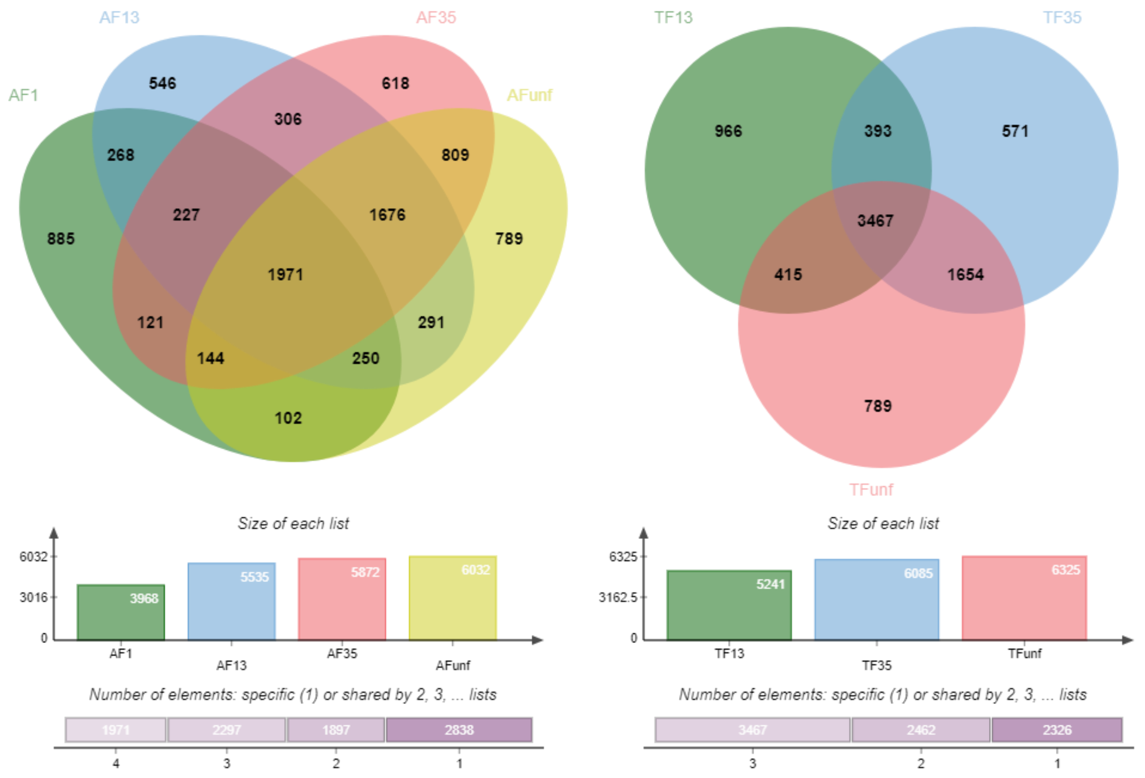

Figure S11: Venn diagrams showing shared peptides between fractions of the AF PPH (A) and the TF PPH (B).

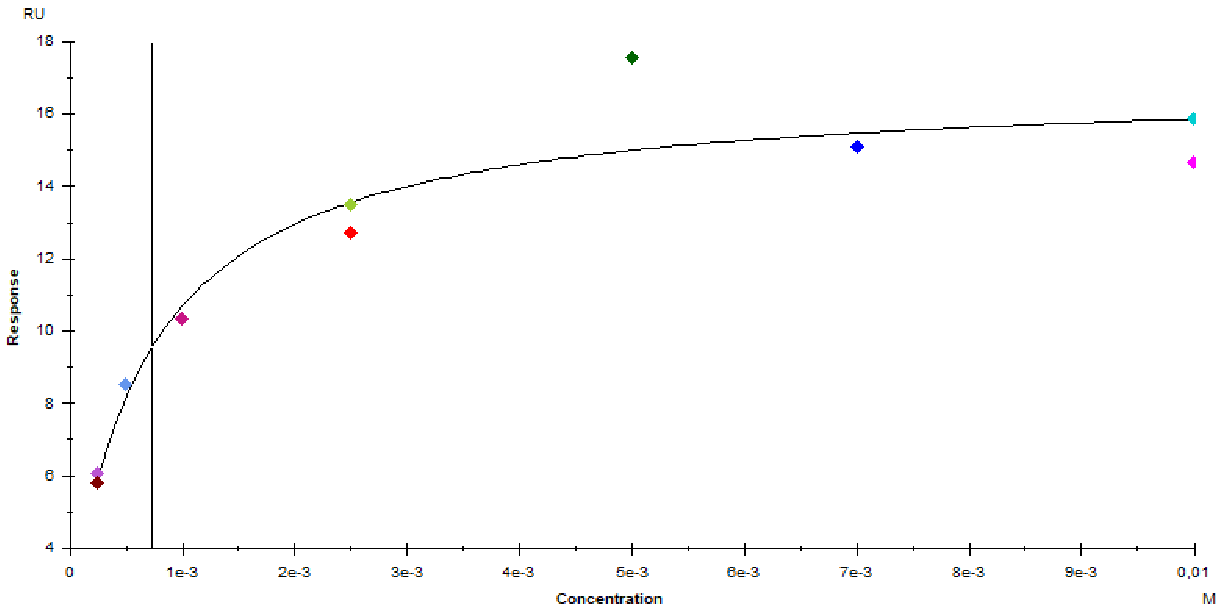

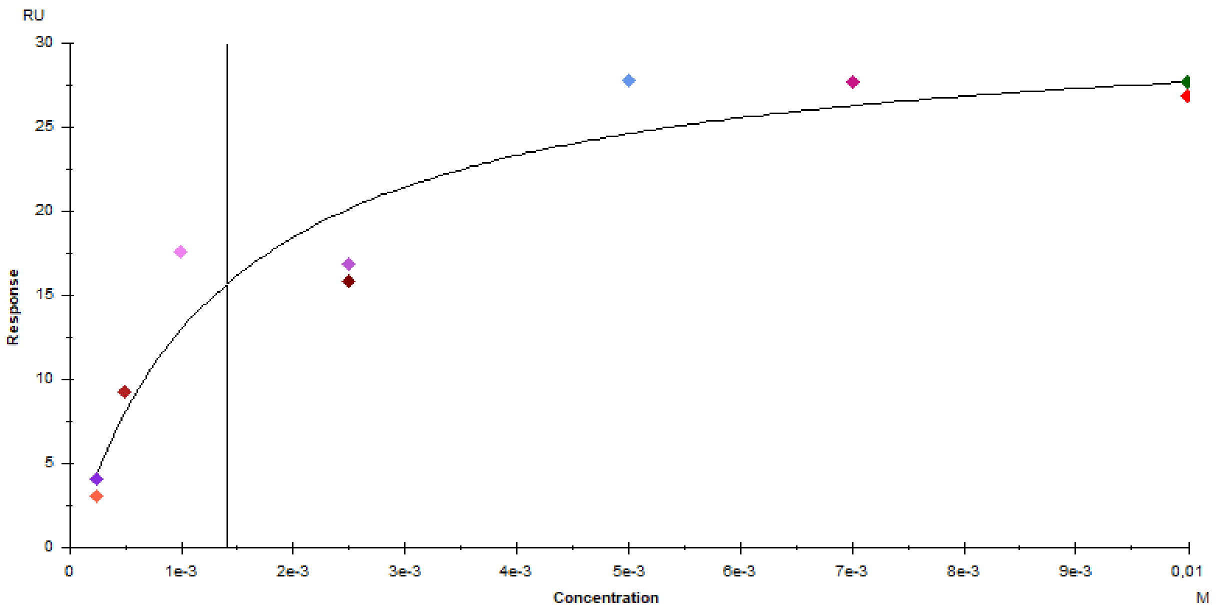
RU
Response
Concentration
M

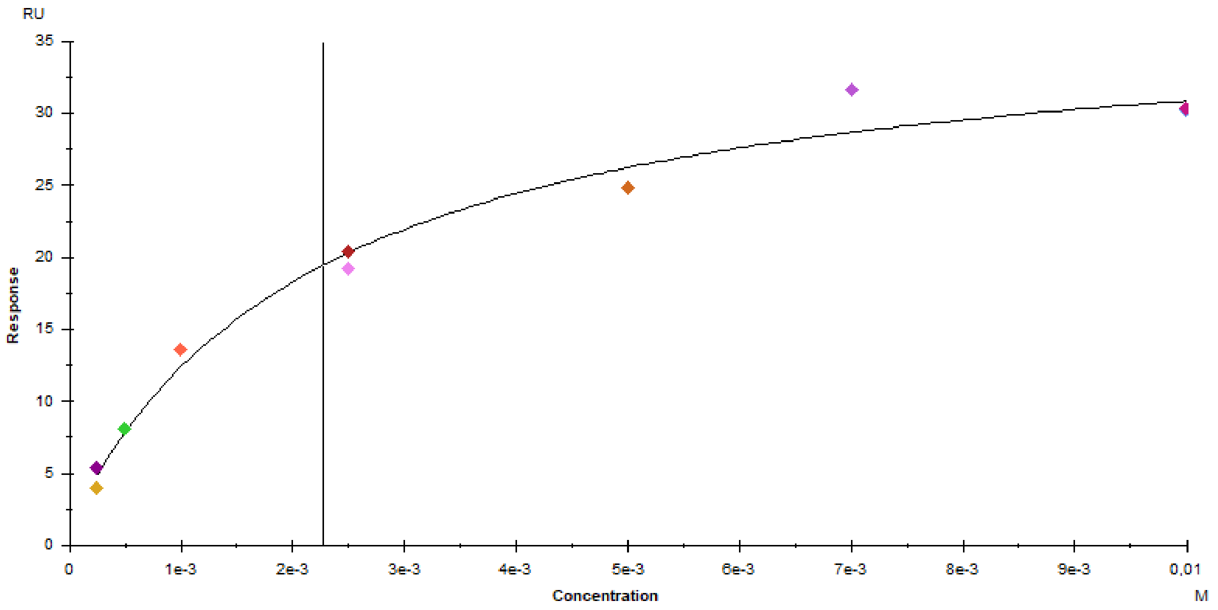
RU
Response
Concentration
M

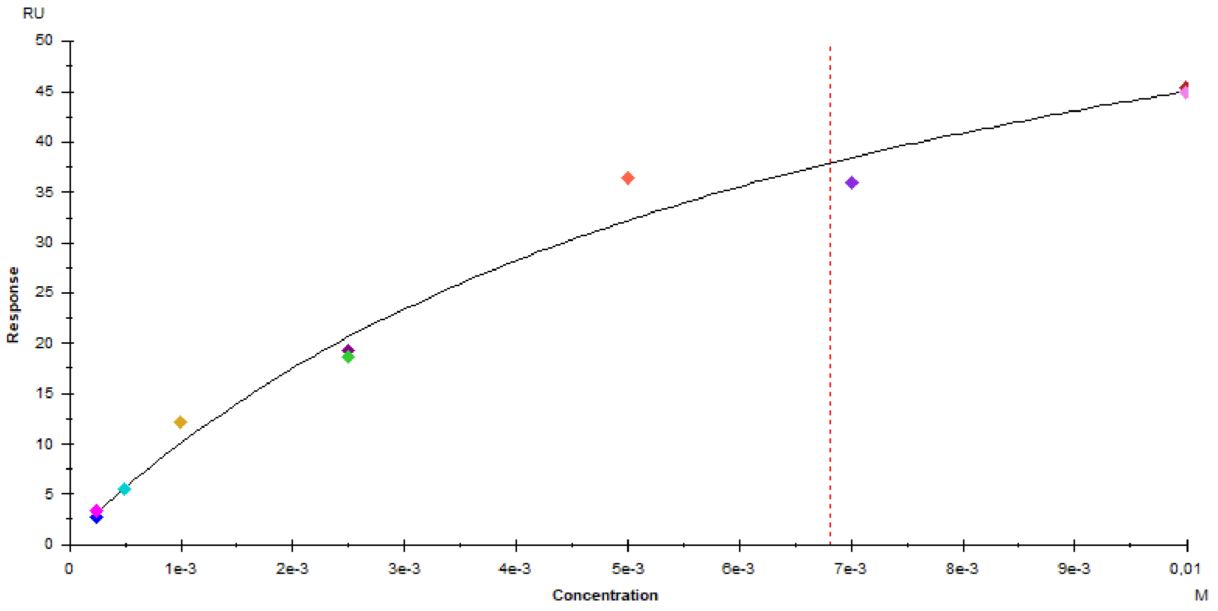
RU
Response
Concentration
M

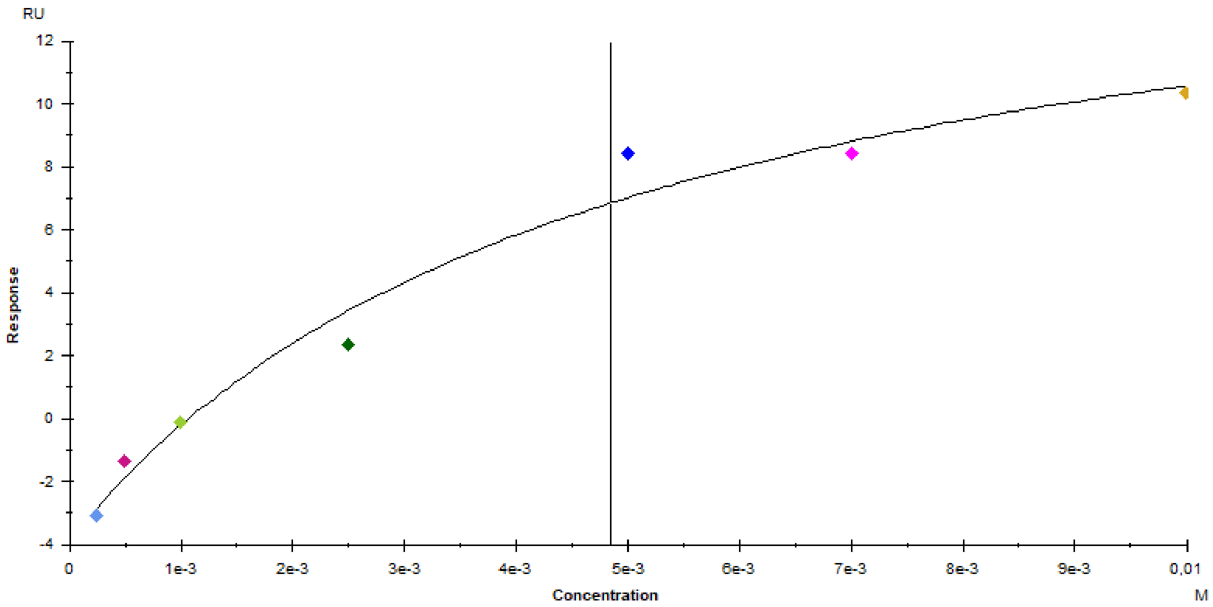

RU
Response
Concentration
M

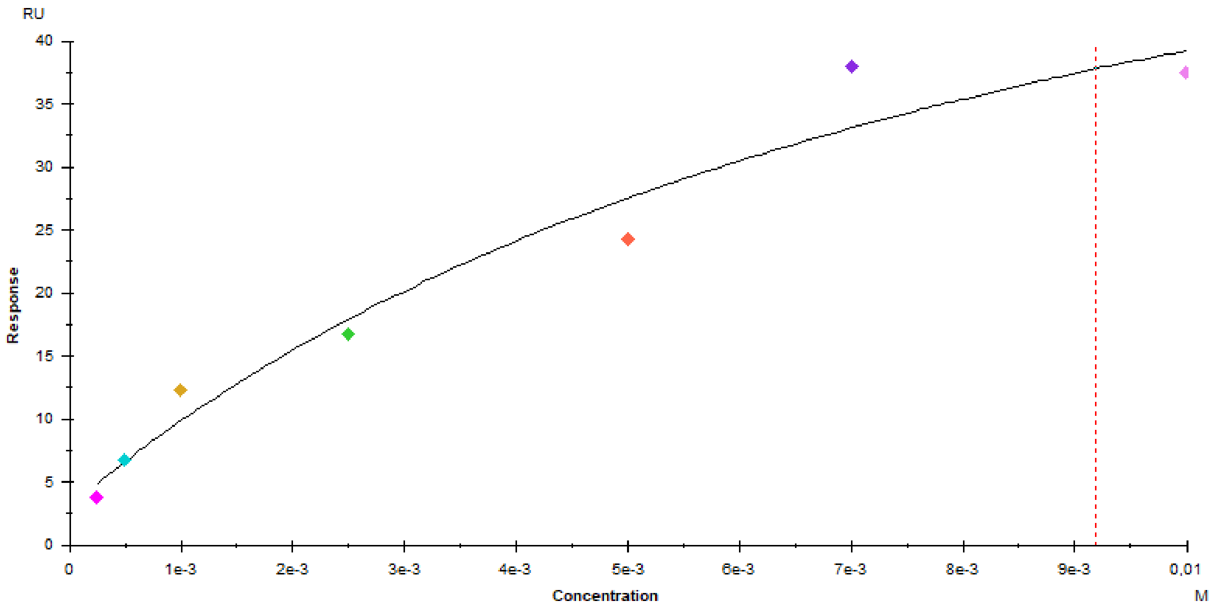

RU
Response
Concentration
M

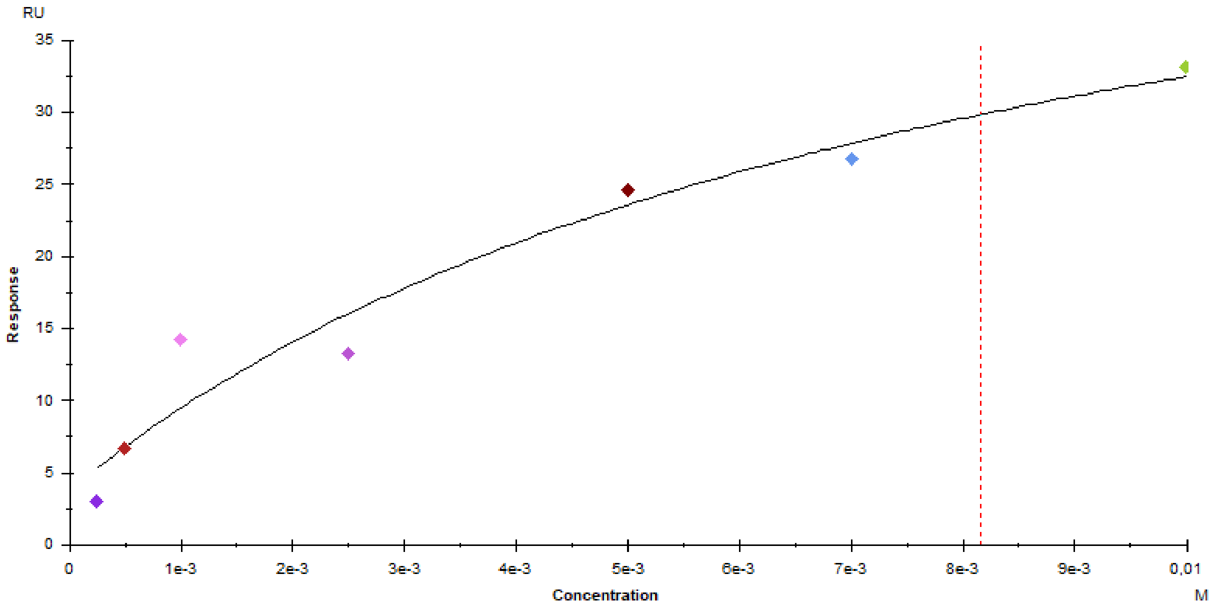

RU
Response
Concentration
M

Figure S12: Surface plasmon resonance sorption isotherms. From top to bottom: AF1, AF13, AF35, AFPPH, TF13, TF35, TFPPH

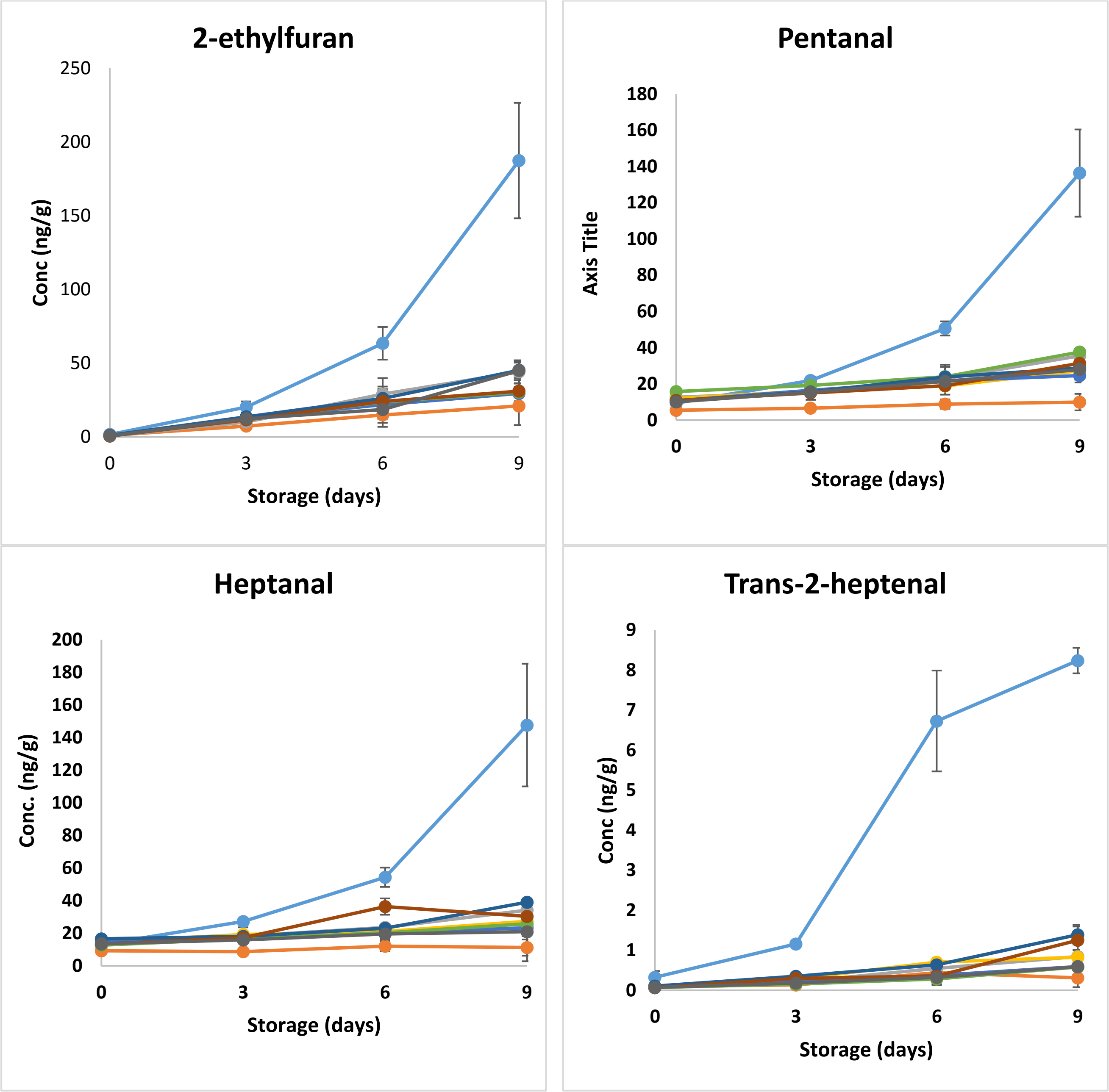

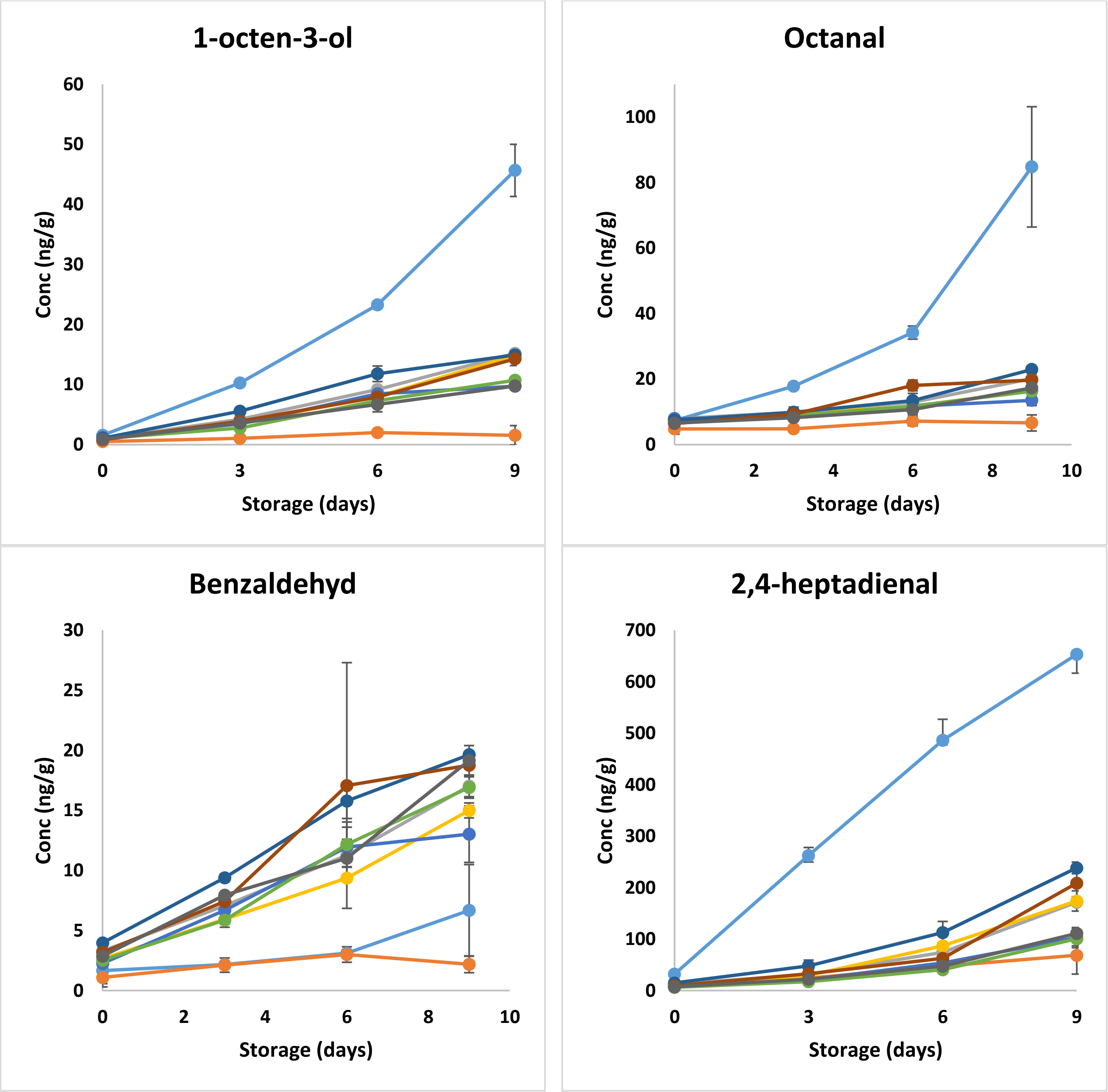
1-octen-3-ol
Conc (ng/g)
60
50
40
30
20
10
0
0
3
6
9
Storage (days)
Octanal
Conc (ng/g)
100
80
60
40
20
0
0
2
4
6
8
10
Storage (days)
Benzaldehyd
Conc (ng/g)
30
25
20
15
10
5
0
0
2
4
6
8
10
Storage (days)
2,4-heptadienal
Conc (ng/g)
700
600
500
400
300
200
100
0
0
3
6
9
Storage (days)

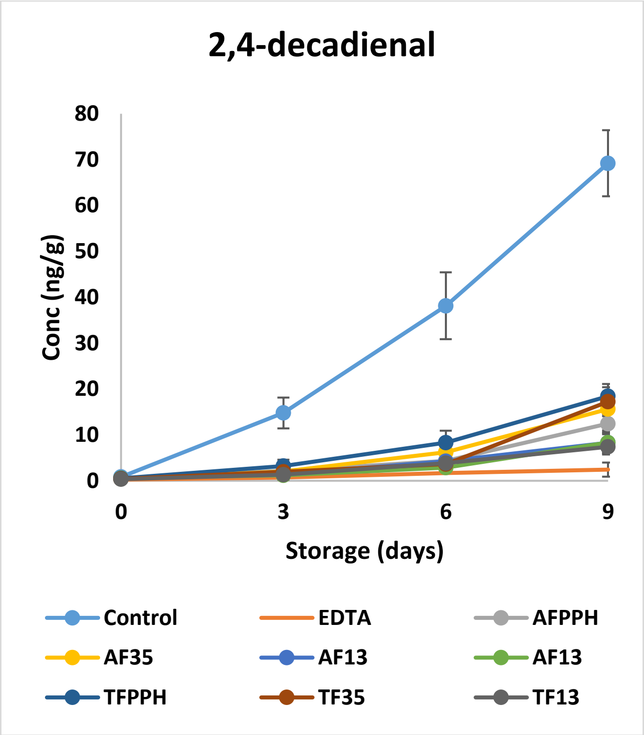

Figure S13: Evolution of SVOPs in 5% fish oil-in-water emulsions during 9 days of storage.
